# Optimized dispersion of nanoparticles for biological *in vitro *and *in vivo *studies

**DOI:** 10.1186/1743-8977-5-14

**Published:** 2008-11-06

**Authors:** Peter Bihari, Minnamari Vippola, Stephan Schultes, Marc Praetner, Alexander G Khandoga, Christoph A Reichel, Conrad Coester, Timo Tuomi, Markus Rehberg, Fritz Krombach

**Affiliations:** 1 Walter Brendel Centre of Experimental Medicine, Ludwig-Maximilians-Universität München, Munich, Germany; 2 Finnish Institute of Occupational Health, Helsinki, Finland; 3 Department of Pharmacy, Pharmaceutical Technology and Biopharmaceutics, Ludwig-Maximilians-Universität München, Munich, Germany

## Abstract

**Background:**

The aim of this study was to establish and validate a practical method to disperse nanoparticles in physiological solutions for biological *in vitro *and *in vivo *studies.

**Results:**

TiO_2 _(rutile) dispersions were prepared in distilled water, PBS, or RPMI 1640 cell culture medium. Different ultrasound energies, various dispersion stabilizers (human, bovine, and mouse serum albumin, Tween 80, and mouse serum), various concentrations of stabilizers, and different sequences of preparation steps were applied. The size distribution of dispersed nanoparticles was analyzed by dynamic light scattering and zeta potential was measured using phase analysis light scattering. Nanoparticle size was also verified by transmission electron microscopy. A specific ultrasound energy of 4.2 × 10^5 ^kJ/m^3 ^was sufficient to disaggregate TiO_2 _(rutile) nanoparticles, whereas higher energy input did not further improve size reduction. The optimal sequence was first to sonicate the nanoparticles in water, then to add dispersion stabilizers, and finally to add buffered salt solution to the dispersion. The formation of coarse TiO_2 _(rutile) agglomerates in PBS or RPMI was prevented by addition of 1.5 mg/ml of human, bovine or mouse serum albumin, or mouse serum. The required concentration of albumin to stabilize the nanoparticle dispersion depended on the concentration of the nanoparticles in the dispersion. TiO_2 _(rutile) particle dispersions at a concentration lower than 0.2 mg/ml could be stabilized by the addition of 1.5 mg/ml albumin. TiO_2 _(rutile) particle dispersions prepared by this method were stable for up to at least 1 week. This method was suitable for preparing dispersions without coarse agglomerates (average diameter < 290 nm) from nanosized TiO_2 _(rutile), ZnO, Ag, SiO_x_, SWNT, MWNT, and diesel SRM2975 particulate matter.

**Conclusion:**

The optimized dispersion method presented here appears to be effective and practicable for preparing dispersions of nanoparticles in physiological solutions without creating coarse agglomerates.

## Background

In recent years, there has been a dramatic increase in research, technology, and production of nanoparticles. Nanoparticles are defined as particles with lengths ranging from 1 to 100 nanometers in two or three dimensions [1]. These nanoscaled particles have physico-chemical properties different from those of bulk material and, thus, offer opportunities for the development of new applications. Some of these engineered nanoparticles are already in use in a diverse array of applications including medicine, food, clothes, personal care products, information technology, and construction materials, resulting in a wide range of exposure scenarios. Therefore, it has become important to determine the potential hazards of nanoparticles on human health. [2-4]

Nanoparticles can come in contact with the human body through inhalation but also through ingestion, dermal deposition, or by medical applications through injection [5]. Nanoparticles having entered the body through inhalation can translocate into the systemic circulation, reach various remote organs, and affect their function [6].

For investigations of the *in vivo *effects of nanoparticles in the circulation and for measuring the effects of nanoparticles on different types of cells *in vitro*, nanoparticles have to be dispersed in physiological solutions. However, particles in solutions with physiological salt concentrations and pH values form micrometer-sized coarse agglomerates [7-9]. Coarse agglomerates of nanoparticles have been shown to exert different biological effects as compared to well-dispersed nanoparticles [9-12]. Therefore, investigating the biological effects of nanoscaled particles with dispersions containing coarse agglomerates is not appropriate. Previously, different methods have been published on how to avoid the formation of coarse agglomerates of nanoparticles dispersed in physiological solutions. The importance of the correct ultrasound energy as well as the use of dispersion stabilizers was emphasized for the optimal deagglomeration of nanoparticles [13]. Pulmonary surfactant, Tween, bronchoalveolar lavage fluid, albumin, or serum were used as dispersion stabilizers in physiological solutions [8,9,11]. Sonication preceding the addition of a dispersion stabilizer to the nanoparticle dispersion has been shown to be more effective than sonication afterwards [7]. However, most of these studies have investigated only one aspect of the particle dispersion method or tested only one nanoparticle type. For practical use in nanotoxicology experiments, a complete method including all these aspects and working on a wide range of different types of nanoparticles is necessary.

Therefore, the aim of our study was to systematically analyze the importance of these aspects for the preparation of nanoparticles and to set up an optimized nanoparticle dispersion method for *in vitro *and *in vivo *studies. For this purpose, we prepared different nanoparticle dispersions, measured the size and zeta potential of the dispersions, and analyzed the samples with transmission electron microscopy. We tested different ultrasound energy levels, distinct dispersion stabilizers (human, bovine, mouse albumin, Tween 80, and mouse serum), various dispersion stabilizer and nanoparticle concentrations, and different sequences of preparation steps. We also tested our method on a broad range of different types of nanoparticles and measured the stability of the dispersion over time.

## Results

### Measurement of polystyrene beads

The accuracy of size distribution and zeta potential measurements of spherical particles was verified by measuring 60–65-nm polystyrene beads with different surface charges. The size measurements demonstrated almost identical results to those reported by the manufacturer. Amine particles had a positive and carboxyl as well as unmodified particles a negative zeta potential (Tab. 1).

**Table 1:** Size and zeta potential of different type of nanoparticles.

*Particle*	*Average diameter (nm)*	*PdI*	*Zeta Potential (mV)*
*TiO*_2_*rutile – Sigma*			
DI H_2_O no US	502 ± 34	0.434 ± 0.086	-44.2 ± 0.3
DI H_2_O	160 ± 2	0.166 ± 0.015	-40.9 ± 3
PBS	641 ± 69	0.263 ± 0.022	-19.5 ± 6.3
PBS, HSA	186 ± 10	0.212 ± 0.03	-8.8 ± 0.9
PBS, Tween	578 ± 132	0.248 ± 0.017	-13.3 ± 4.4
PBS, mserum	175 ± 5	0.270 ± 0.046	-10.7 ± 1.5

*Diesel – SRM 2975*			
DI H_2_O no US	347 ± 21	0.397 ± 0.047	-45.2 ± 2.7
DI H_2_O	144 ± 1	0.132 ± 0.009	-48.4 ± 1.7
PBS	684 ± 284	0.249 ± 0.064	-32.0 ± 2.8
PBS, HSA	163 ± 3	0.152 ± 0.008	9.6 ± 0.6
PBS, Tween	151 ± 1	0.143 ± 0.012	-7.0 ± 0.2
PBS, mserum	168 ± 13	0.209 ± 0.033	-9.2 ± 1.4

*Silver*			
DI H_2_O no US	403 ± 125	0.455 ± 0.039	-20.1 ± 3.9
DI H_2_O	161 ± 12	0.338 ± 0.055	-29.8 ± 0.3
PBS	223 ± 8	0.343 ± 0.026	-24.5 ± 3.1
PBS, HSA	172 ± 22	0.343 ± 0.018	-11.3 ± 0.3
PBS, Tween	194 ± 23	0.368 ± 0.014	-9.8 ± 1.9
PBS, mserum	158 ± 5	0.305 ± 0.063	-11.0 ± 0.6

*TiO*_2_*anatase*			
DI H_2_O no US	1169 ± 48	0.462 ± 0.02	13.8 ± 2.1
DI H_2_O	517 ± 60	0.431 ± 0.055	-18.7 ± 0.4
PBS	890 ± 230	0.369 ± 0.077	-23.0 ± 1.7
PBS, HSA	521 ± 25	0.475 ± 0.023	-9.6 ± 1.1
PBS, Tween	818 ± 11	0.358 ± 0.029	-14.0 ± 2.5
PBS, mserum	574 ± 92	0.474 ± 0.111	-10.7 ± 1.1

*ZnO*			
DI H_2_O no US	1298 ± 252	0.721 ± 0.072	10.6 ± 2.0
DI H_2_O	278 ± 72	0.414 ± 0.086	-29.4 ± 6.0
PBS	517 ± 174	0.445 ± 0.053	-29.3 ± 2.8
PBS, HSA	267 ± 6	0.288 ± 0.059	-11.6 ± 0.6
PBS, Tween	457 ± 135	0.360 ± 0.041	-14.6 ± 7.3
PBS, mserum	190 ± 8	0.544 ± 0.103	-7.7 ± 1.1

*SiOx*			
DI H_2_O no US	1121 ± 304	0.593 ± 0.014	-33.4 ± 1.8
DI H_2_O	370 ± 49	0.488 ± 0.047	-21.1 ± 19.8
PBS	852 ± 267	0.617 ± 0.025	-14.5 ± 0.2
PBS, HSA	251 ± 27	0.880 ± 0.107	-10.4 ± 0.7
PBS, Tween	398 ± 51	0.532 ± 0.040	-3.7 ± 0.4
PBS, mserum	132 ± 49	0.497 ± 0.192	-11.1 ± 0.6

*SWNT*			
DI H_2_O no US	689 ± 111	0.569 ± 0.100	-7.0 ± 7.6
DI H_2_O	372 ± 59	0.560 ± 0.076	-23.1 ± 10.4
PBS	977 ± 46	0.526 ± 0.131	-2.6 ± 2.3
PBS, HSA	285 ± 79	0.605 ± 0.143	-10.1 ± 0.7
PBS, Tween	291 ± 89	0.531 ± 0.103	-8.5 ± 3.3
PBS, mserum	115 ± 51	0.666 ± 0.132	-7.7 ± 1.6

*MWNT*			
DI H_2_O no US	309 ± 48	0.304 ± 0.027	-2.7 ± 2.2
DI H_2_O	262 ± 101	0.397 ± 0.048	-22.5 ± 19.2
PBS	486 ± 173	0.424 ± 0.079	-19.6 ± 5.8
PBS, HSA	269 ± 56	0.406 ± 0.027	-9.7 ± 1.1
PBS, Tween	206 ± 13	0.292 ± 0.037	-4.8 ± 0.5
PBS, mserum	166 ± 10	0.415 ± 0.006	-7.8 ± 0.4

*Plain polystyrene beads*			
DI H_2_O no US	67 ± 0.4	0.030 ± 0.004	-57.0 ± 5.3
PBS no US	73 ± 7	0.135 ± 0.068	-24.8 ± 5.7

*Carboxyl modified polystyrene beads*			
DI H_2_O no US	60 ± 0.3	0.046 ± 0.021	-56.7 ± 0.3
PBS no US	58 ± 0.9	0.057 ± 0.018	-32.5 ± 2.5

*Amine modified polystyrene beads*			
DI H_2_O no US	68 ± 0.5	0.062 ± 0.035	59.7 ± 4.9
PBS no US	86 ± 8	0.204 ± 0.029	19.0 ± 3.4

Nanoparticles were prepared in distilled water at a concentration of 0.02 mg/ml with (DI H2O) or without (DI H2O no US) sonication. For other measurements, nanoparticles were prepared in distilled water at a concentration of 0.02 mg/ml by sonication and addition of human serum albumin (PBS, HSA), Tween 80 (PBS, Tween), mouse serum (PBS, mserum) or distilled water (PBS) previous to addition of PBS. The measurements were made in triplicate. The average value and the standard deviation of the measurements are shown.

### Ultrasound energy

**Figure 1:**
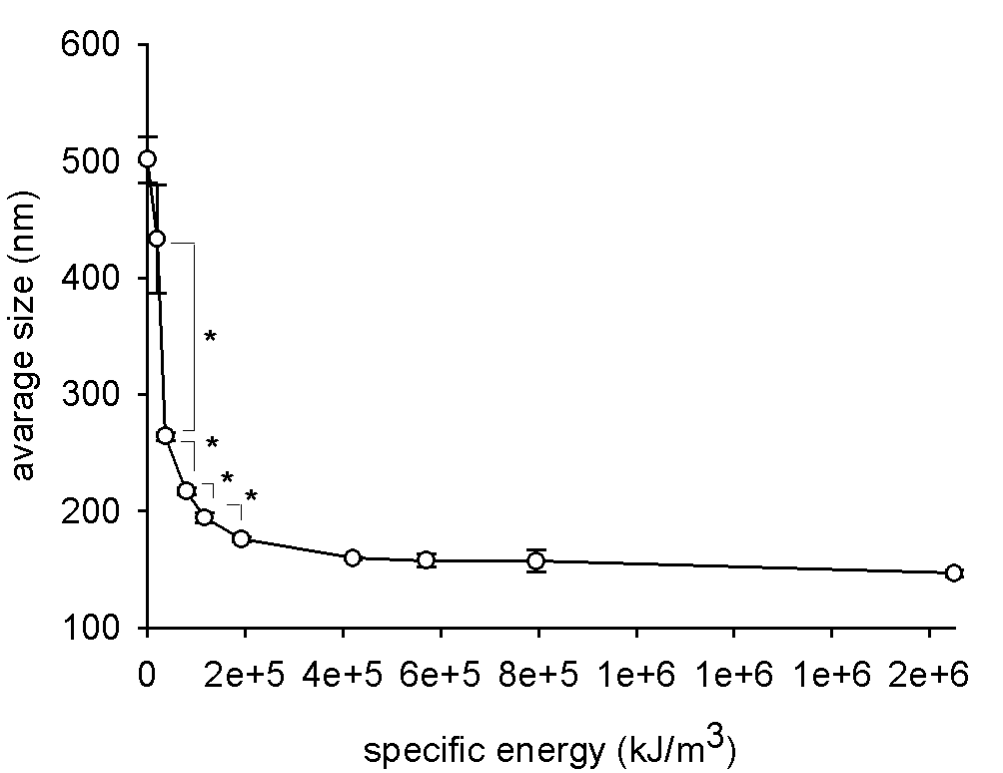
**Effect of intensity of sonication on TiO_2 _(rutile) particle size**. TiO_2 _(rutile) dispersed in distilled water at a concentration of 0.02 mg/ml was sonicated with different specific energies, and the average of the hydrodynamic diameter of the particles was measured. The experiments were carried out in triplicates (*, p < 0.05).

The effects of different ultrasound energies were tested on TiO_2 _(rutile) suspensions in distilled water. A specific ultrasound energy of 4.2 × 10^5 ^kJ/m^3 ^(power consumption: 7 W, 1 mL dispersion, 60 sec sonication) was sufficient to disaggregate TiO_2 _(rutile) nanoparticles as indicated by the reduction of the particle diameter from 527.6 ± 34.2 to 159.7 ± 2.3 nm (Fig. 1) and of the polydispersity index (PdI, describes the width of the particle size distribution) from 0.434 ± 0.086 to 0.166 ± 0.015 (data not shown). Higher energy input did not further improve size reduction (Fig. 1).

### Sequence of preparation steps

**Figure 2:**
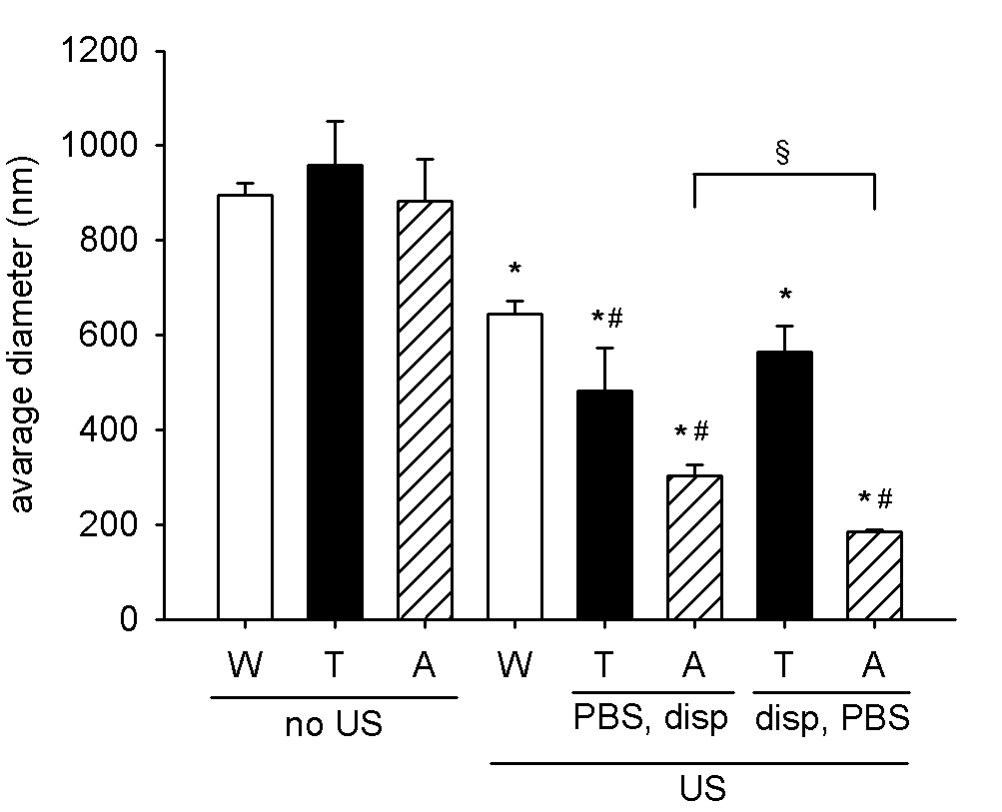
**Role of the sequence of preparation steps**. TiO_2 _(rutile) dispersed in distilled water at a concentration of 0.02 mg/ml was sonicated with 4.2 × 10^5 ^kJ/m^3 ^specific energy (US) or not sonicated (no US). Tween 80 0.1% (T), HSA 1.5 mg/ml (A) or distilled water (W) was given to the dispersion before (disp, PBS) or after (PBS, disp) addition of concentrated PBS. The average hydrodynamic diameter of the particles was measured (n = 4; *, p < 0.05 vs. dispersion in distilled water (W) without sonication (no US), #, p < 0.05 vs. dispersion in distilled water (W) with sonication (US), § p < 0.05).

**Figure 3:**
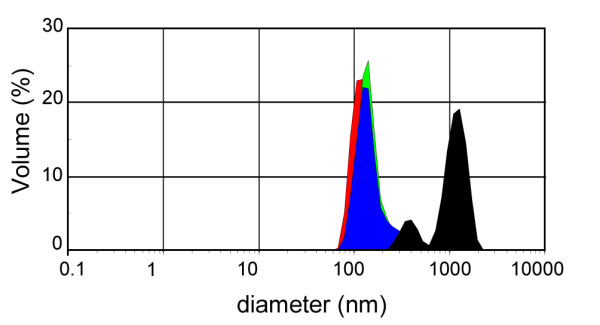
**Size distribution by volume of a TiO_2 _(rutile) dispersion measured after each preparation step**. TiO_2 _(rutile) was dispersed in distilled water and sonicated (red), then HSA (blue) and finally concentrated PBS (green) was given to the dispersion. TiO_2 _(rutile) was also prepared on the same way but without HSA (black).

**Figure 4:**
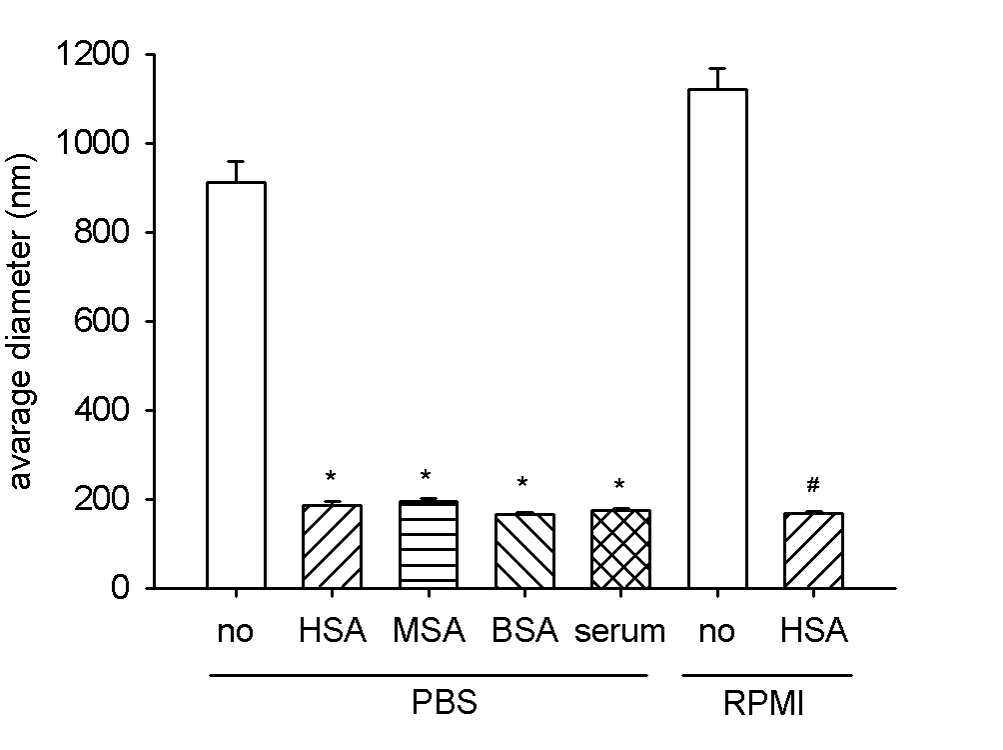
**Albumin from different species and serum as dispersion stabilizer**. TiO_2 _(rutile) dispersed in distilled water at a concentration of 0.02 mg/ml was sonicated, and HSA, MSA, BSA or mouse serum was given to the dispersion before the addition of concentrated PBS. The average hydrodynamic diameter of the particles was measured. The experiments were carried out in triplicates (*, p < 0.05 vs. dispersion without albumin in PBS; #, p < 0.05 vs. dispersion without albumin in RPMI).

Different sequences of preparation steps of particle dispersions were assessed on TiO_2 _(rutile) (Fig. 2). TiO_2 _(rutile) dispersed in phosphate buffered saline (PBS) had a higher average diameter (912.1 ± 47.5 nm) and a greater PdI (0.509 ± 0.017) than when dispersed in distilled water. Human serum albumin (HSA) or Tween 80 added to the TiO_2 _(rutile) dispersion without previous sonication did not reduce the particle diameter in PBS. However, the TiO_2 _(rutile) particle diameter was reduced in PBS when HSA or Tween 80 was added to the dispersion after sonication. In the case of HSA, the diameter of TiO_2 _(rutile) particles was further reduced when the stabilizer was added prior to addition of PBS. Best results were obtained with HSA as dispersion stabilizer when we first sonicated the TiO_2 _(rutile) nanoparticles in distilled water, then added the dispersion stabilizer, and at the end added buffered salt solution to the dispersion (average diameter = 186.4 ± 9.9 nm, PdI = 0.212 ± 0.03). In this case, the addition of HSA prior to the addition of PBS prevented the TiO_2 _(rutile) particles deagglomerated by sonication from reagglomeration (Fig. 3). Interestingly, the diameter of TiO_2 _(rutile) particles was slightly but significantly elevated after HSA addition (from 159.7 ± 2.3 to 174.2 ± 2.2, Fig. 3). This dispersion protocol also worked well when RPMI 1640 cell culture medium was used as a dispersion medium. Similar results where obtained using bovine serum albumin (BSA), mouse serum albumin (MSA), or mouse serum as dispersion stabilizers (Fig. 4).

### Albumin and nanoparticle concentration

**Figure 5:**
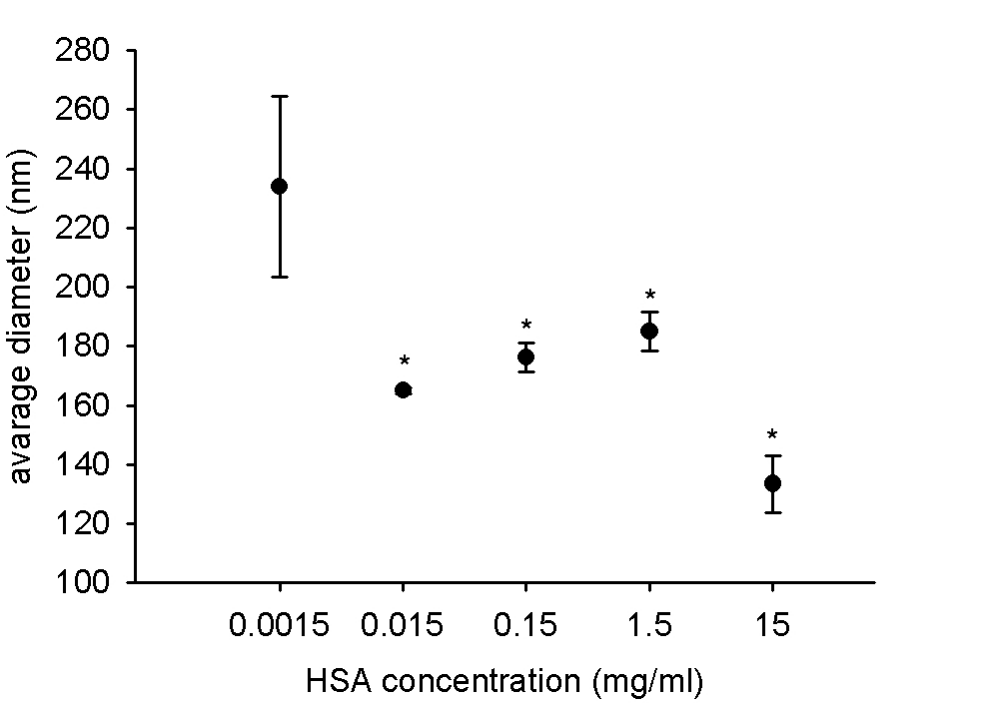
**TiO_2 _(rutile) particle size in dispersions with different HSA concentrations**. TiO_2 _(rutile) dispersed in distilled water at a concentration of 0.02 mg/ml was sonicated and HSA at concentrations ranging from 0.0015 to 15 mg/ml was given to the dispersion prior to addition of concentrated PBS. The average hydrodynamic diameter of the particles was measured. The experiments were carried out in triplicates (*, p < 0.05 vs. dispersion with 0.0015 mg/ml HSA).

**Figure 6:**
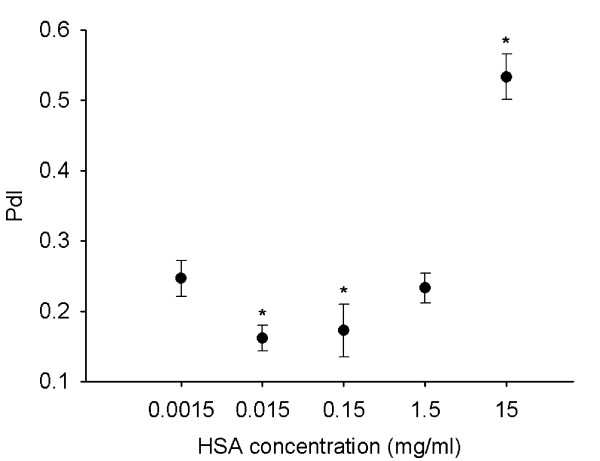
**Polydispersity index of TiO_2 _(rutile) in dispersions with different HSA concentrations**. TiO_2 _(rutile) dispersed in distilled water at a concentration of 0.02 mg/ml was sonicated and HSA at concentrations ranging from 0.0015 to 15 mg/ml were given to the dispersion prior to addition of concentrated PBS. Polydispersity index (PdI) of the particles was measured. The experiments were carried out in triplicates (*, p < 0.05 vs. dispersion with 0.0015 mg/ml HSA).

To optimize the HSA concentration for stabilization of dispersions, we prepared dispersions with different HSA or TiO_2 _(rutile) concentrations. When changing HSA concentration at a constant TiO_2 _(rutile) concentration (0.02 mg/ml), we found that a HSA concentration between 0.015 mg/ml and 15 mg/ml prevented the formation of coarse TiO_2 _(rutile) agglomerates. However, at a HSA concentration of 0.0015 mg/ml, the TiO_2 _(rutile) average diameter and the PdI value were increased (Fig. 5 and Fig. 6). At a HSA concentration of 15 mg/ml, the average diameter of TiO_2 _(rutile) particles was slightly decreased. This decrease is not the result of a real change in the TiO_2 _(rutile) particle size, but the consequence of the presence of particles with a diameter of 7.1 ± 0.1 nm (data not shown) in the dispersion. The presence of these particles, corresponding to free HSA molecules, causes a shift of the average diameter. The presence of the two types of particles with different diameters (TiO_2 _and free HSA molecules) in the dispersion can also be seen in the strong increase of the PdI.

**Figure 7:**
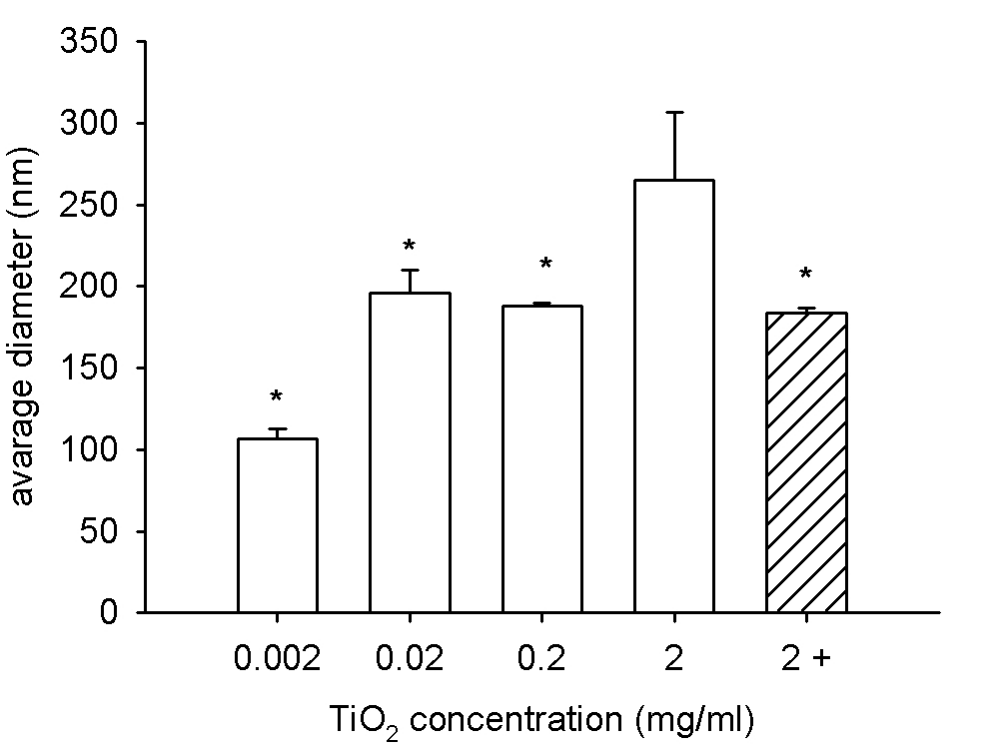
**TiO_2 _(rutile) particle size in dispersions with different TiO_2 _concentrations**. TiO_2 _(rutile) dispersed in distilled water at concentrations ranging from 0.002 to 2 mg/ml was sonicated and 1.5 mg/ml HSA was given to the dispersion before adding concentrated PBS to the dispersion. TiO_2 _(rutile) dispersions at a concentration of 2 mg/ml were also prepared in the same way but with addition of 10 times more (15 mg/ml) HSA (hatched bar). The average hydrodynamic diameter of the particles was measured. The experiments were carried out in triplicates (*, p < 0.05 vs. dispersion with 2 mg/ml TiO_2 _(rutile)).

**Figure 8:**
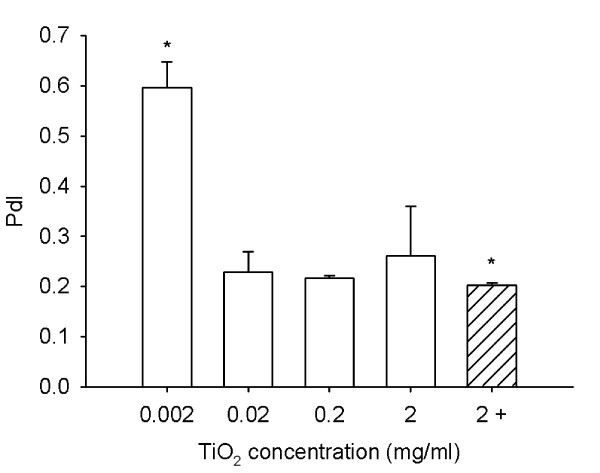
**Polydispersity index of TiO_2 _(rutile) dispersions with different TiO_2 _concentrations**. TiO_2 _(rutile) dispersed in distilled water at concentrations ranging from 0.002 to 2 mg/ml was sonicated and 1.5 mg/ml HSA was given to the dispersion before adding concentrated PBS to the dispersion. TiO_2 _(rutile) dispersions at a concentration of 2 mg/ml were also prepared in the same way but with addition of 10 times more (15 mg/ml) HSA (hatched bar). Polydispersity index (PdI) of the particles was measured. The experiments were carried out in triplicates (*, p < 0.05 vs. dispersion with 2 mg/ml TiO_2 _(rutile)).

Next, we prepared dispersions with different TiO_2 _(rutile) concentrations, but the same HSA (1.5 mg/ml) concentration. We found that HSA prevented the formation of coarse agglomerates at TiO_2 _(rutile) concentrations ranging from 0.002 to 0.2 mg/ml. As in the highest HSA concentration, the presence of particles with about 7 nm caused a shift in the average diameter at the lowest TiO_2 _(rutile) concentration. At a TiO_2 _(rutile) concentration of 2 mg/ml, the average diameter and the PdI value were increased. However, this increase in particle size could be avoided by increasing the amount of HSA in the dispersion by 10 times (Fig. 7 and 8).

### Stability

**Figure 9:**
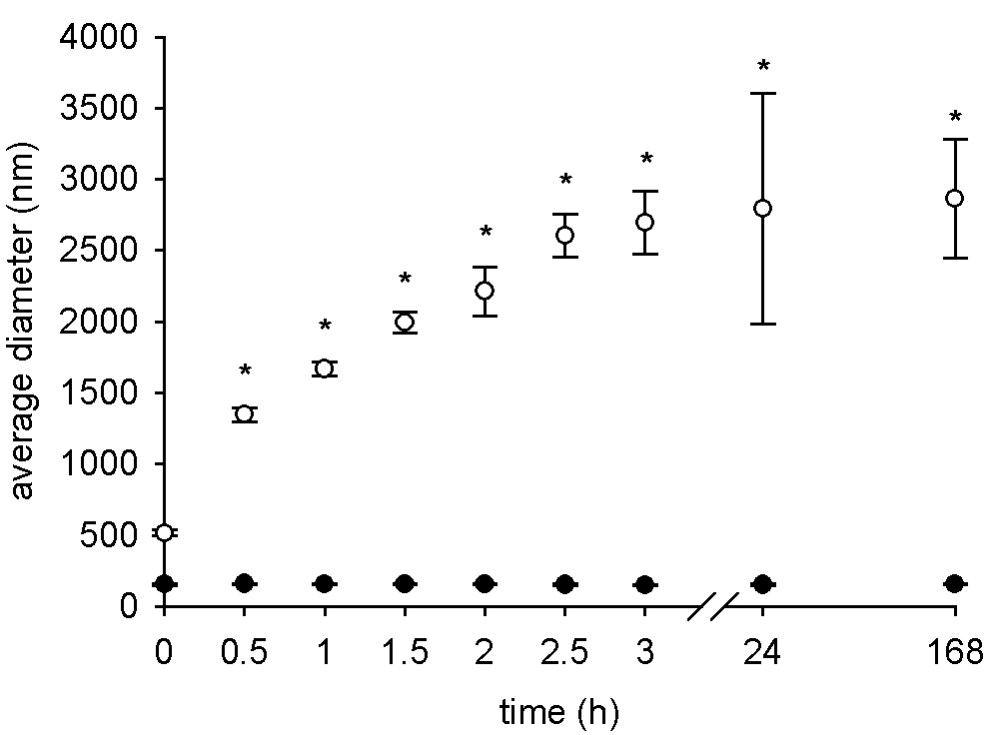
**Stability of TiO_2 _(rutile) dispersion**. TiO_2 _(rutile) dispersions were prepared in distilled water at a concentration of 0.02 mg/ml with (closed circles) or without (open circles) addition of HSA before giving concentrated PBS to the dispersion. The average hydrodynamic diameter of the particles was measured at different time points for up to one week. The experiments were carried out in triplicates (*, p < 0.05, dispersions with vs. without HSA at the same time point).

The stability of the TiO_2 _(rutile) dispersions prepared with 1.5 mg/ml HSA was determined over a time period of one week (Fig. 9). During this time period, the TiO_2 _(rutile) PBS dispersions prepared with HSA remained stable without coarse agglomerates, whereas in dispersions without HSA the average diameter and the PdI values were continually increasing and approaching a plateau at 24 hours.

### Different type of nanoparticles

To test the applicability of our optimized method for other nanoparticles, we prepared and measured TiO_2 _(anatase), ZnO, SWNT, MWNT, Silver, SiO_x_, and nanosized diesel particulate matter using HSA, Tween 80, or mouse serum as dispersion stabilizers (Tab. 1). In all dispersions, the average diameter of the particles was greater then the size of the primary particles, indicating the presence of some agglomerates. For all nanoparticles tested, the addition of HSA, Tween 80, or mouse serum resulted in a decreased average diameter. With HSA as dispersion stabilizer, the average diameter of TiO_2 _(rutile), ZnO, SWNT, MWNT, Silver, SiO_x_, and diesel nanoparticles was below 290 nm. For TiO_2 _(anatase) particles, our method was less effective as indicated by a higher average diameter of the particles. In dispersions of SWNT, MWNT, and SiO_x_nanoparticles, the PdI value was rather high.

### Zeta-potential

All particles had a negative zeta potential in distilled water after sonication (Table 1). Interestingly, ZnO and TiO_2 _(anatase) had a positive zeta-potential upon dispersion in distilled water and became negative after sonication. The particles were less negative when prepared with HSA, Tween 80, or serum in PBS. TiO_2 _(rutile) prepared with HSA in RPMI cell culture medium had a positive zeta potential.

### Transmission electron microscopy

**Figure 10:**
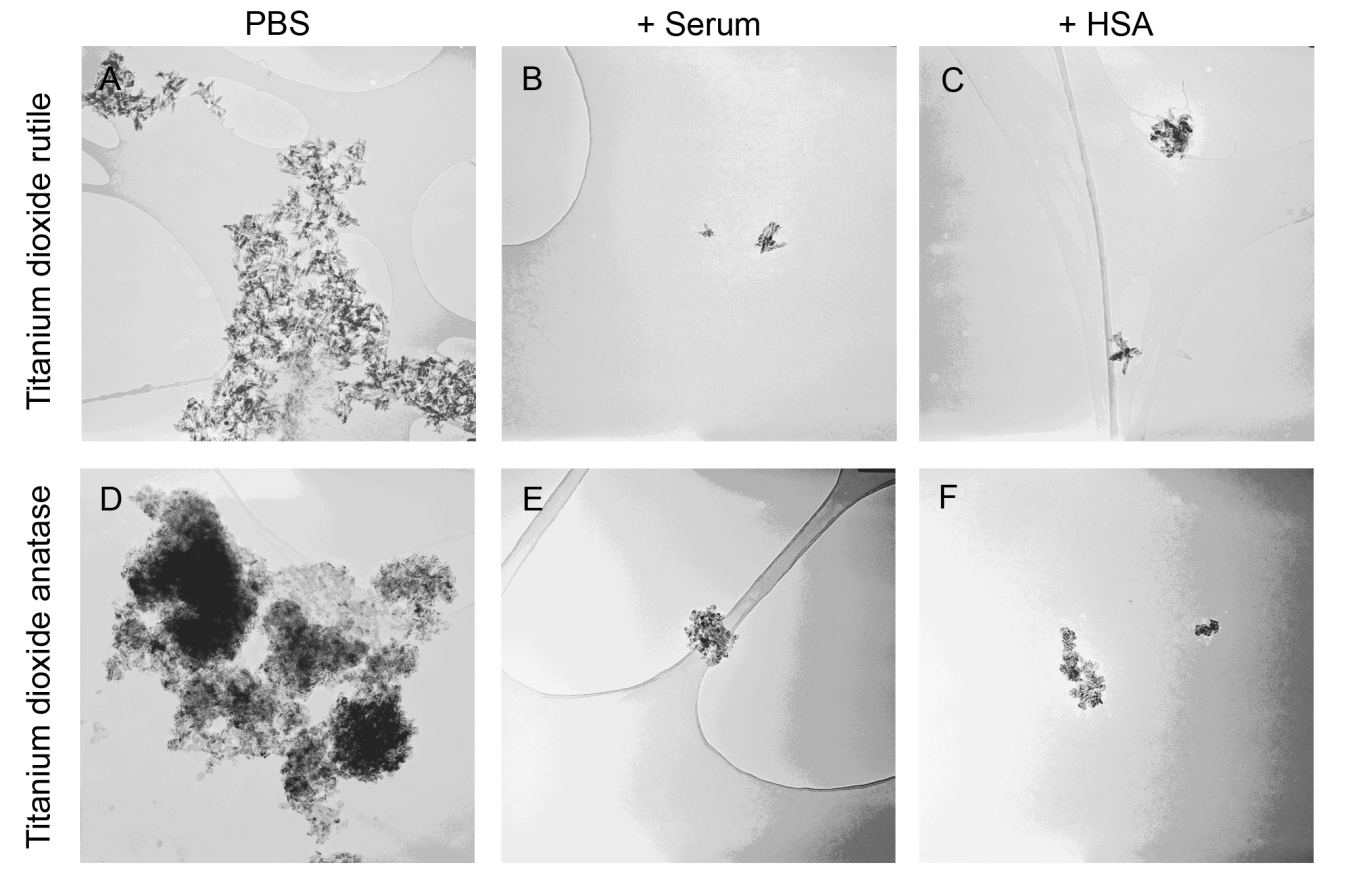
**Electron microscopy of titanium dioxide nanoparticles**. Electron microscopic image at 100,000 times magnification (950 × 950 nm) from TiO_2 _(rutile) and TiO_2 _(anatase) nanoparticle dispersions prepared in distilled water at a concentration of 0.02 mg/ml without stabilizer (PBS) or with addition of human serum albumin (+HSA) or mouse serum (+Serum) before giving concentrated PBS to the dispersion.

**Figure 11:**
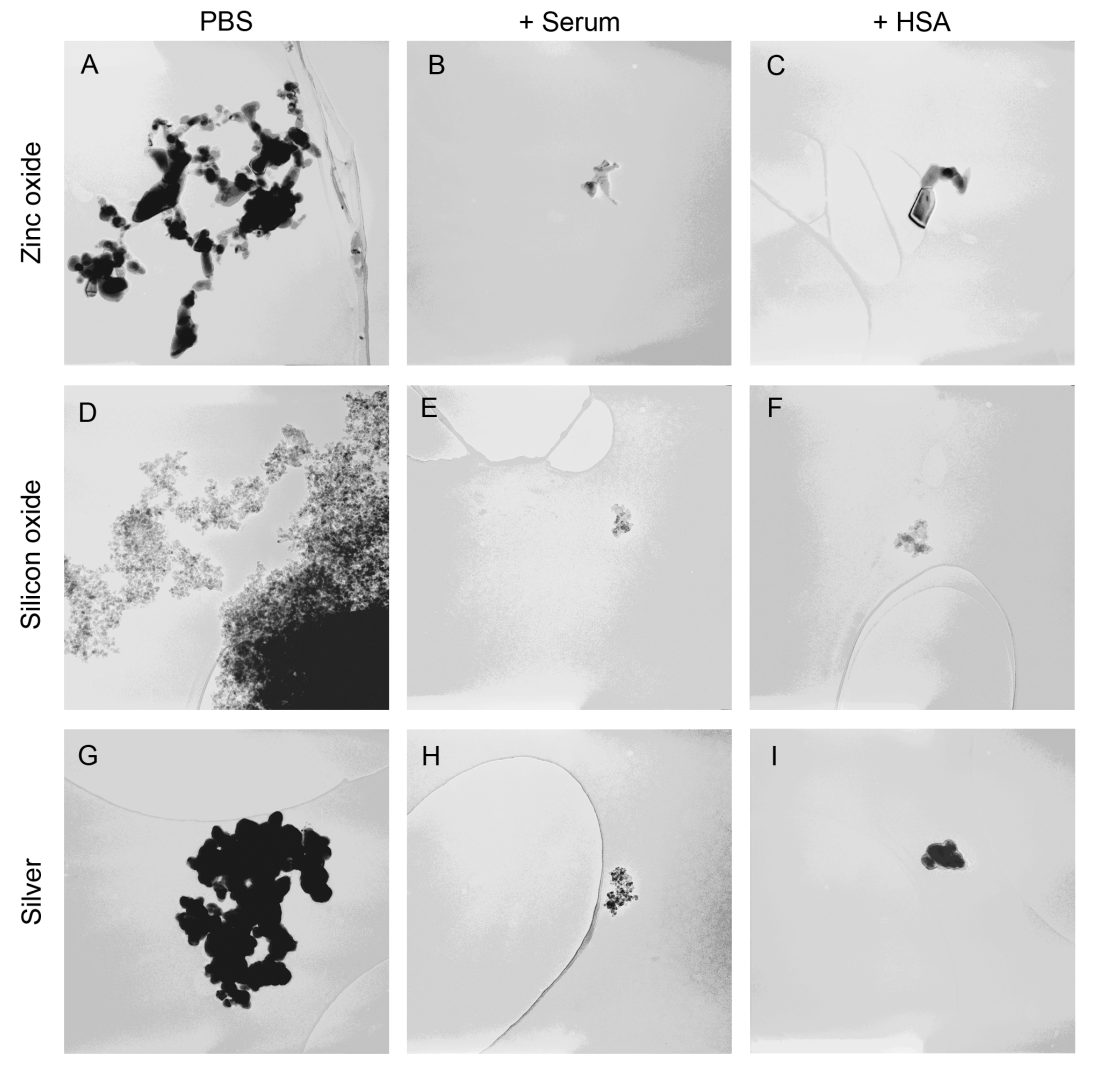
**Electron microscopy of zinc oxide, silicon oxide and silver nanoparticles**. Electron microscopic image at 100,000 times magnification (950 × 950 nm) from ZnO, SiO_x _and silver nanoparticle dispersions prepared in distilled water at a concentration of 0.02 mg/ml without stabilizer (PBS) or with addition of human serum albumin (+HSA) or mouse serum (+Serum) before giving concentrated PBS to the dispersion.

**Figure 12:**
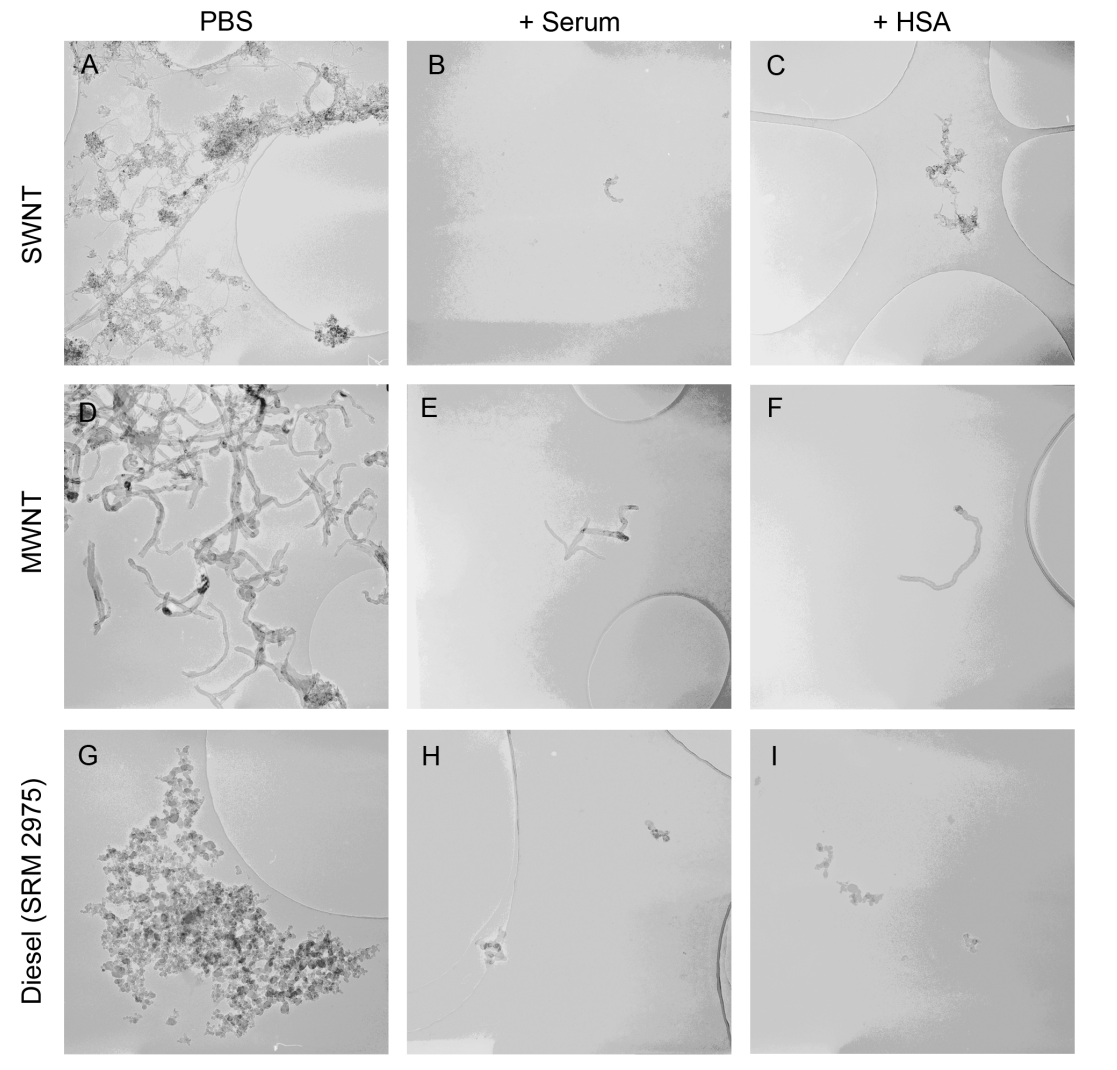
**Electron microscopy of nanotubes and diesel particles**. Electron microscopic image at 100,000 times magnification (950 × 950 nm) from SWNT, MWNT and diesel (SRM 2975) particle dispersions prepared in distilled water at a concentration of 0.02 mg/ml without stabilizer (PBS) or with addition of mouse serum (+Serum) or human serum albumin (+HSA) before giving concentrated PBS to the dispersion.

To visualize nanoparticles in dispersions, we used transmission electron microscopy (TEM). Similarly to the dynamic light scattering measurements, TEM images showed smaller nanoparticle agglomerates in dispersions prepared with the addition of HSA or mouse serum as stabilizers (Fig. 10, 11, 12). In the case of TiO_2 _(rutile) dispersions prepared with HSA, we measured the particle size with an image analyzing software. The mean particle size was 134 ± 71 nm.

## Discussion

The aim of our study was to establish a method for the preparation of stable nanoparticle dispersions, i.e. dispersions without coarse agglomerates in physiological solutions. The stability of particle dispersions depends on the balance between attractive and repulsive forces between the particles (DLVO theory)[14]. In principle, there are two ways to prepare stable dispersions: electrostatic and steric stabilization. With electrostatic stabilization, the zeta potential of the particles provides the repulsive force. In practice, if the zeta potential of the particles is higher than 30 mV or lower than -30 mV the dispersion is stable. The zeta potential of the particles, however, strongly depends on the pH and the electrolyte concentration of the dispersion [14]. At physiological pH and electrolyte concentration, the zeta potential of the particles is not high enough to stabilize the dispersion, and the nanoparticles form coarse agglomerates as it has been shown in this as well as in previous studies [7-9]. Therefore, steric stabilization is used for nanoparticle dispersion stabilization in physiological solutions, where a stabilizer is added to the dispersion adsorbing onto the particle surfaces and preventing them from coming close to one another [15]. This strategy has been shown to be beneficial, and addition of different stabilizers has been demonstrated to decrease the formation of coarse agglomerates and to improve the stability of dispersions [8,9,11]. In our study, we intended to optimize the factors that may influence the effectiveness of steric stabilization, such as sonication, sequence of preparation steps, and concentration of the stabilizer.

To analyze the effectiveness of steric stabilization, i.e measure the size of agglomerates in the dispersion, we used dynamic light scattering that is a reliable technique for measuring particle size and size distribution of dispersed spherical nanomaterials [15]. Moreover, we measured the zeta potential of the particles and visualized the dispersed particles using transmission electron microscopy.

First, we measured the effect of different sonication energies. The result of sonication, e.g. particle size reduction, depends on the applied energy per volume of the dispersion (specific energy) [16,17]. We found that after rapid initial size reduction, a further increase in the specific energy did not lead to a further reduction of the particle size. Thus, a specific energy of 4.2 × 10^5 ^kJ/m^3 ^was optimal for deagglomerating the nanoparticles. Our findings confirm previous observations by Mandzy et al [13].

In a next set of experiments, we analyzed the optimal sequence of particle preparation. The best sequence of dispersion preparation was when we first sonicated the nanoparticles, than added HSA as stabilizer, and finally added PBS to the dispersion. With this dispersion sequence, addition of HSA molecules after sonication prevents the particles from the reagglomeration process caused by the pH and ionic strength of PBS. Similar results were published for the preparation of dispersions of C60 nanoparticles [7]. The stabilizing effect of albumin was found to be species-independent as the particles in the dispersions with HSA, MSA, and BSA had comparable average diameters and were medium-independent since HSA worked in PBS as well as in RPMI cell culture medium.

When HSA molecules are added to the nanoparticles, they adhere to the surface of the particles as indicated by a significant increase in the average diameter of the particles. This finding is in agreement with the prevailing opinion about the formation of a "nanoparticle-protein corona" upon incubation of nanoparticles with proteins [18]. Interestingly, in our measurements, the difference of average particle size between dispersions with and without HSA was 14.5 nm, consistent with approximately twice the diameter of one HSA molecule (7.1 ± 0.1 nm, our measurement with DLS). These data suggest that the HSA molecules may completely cover the nanoparticles. This finding is corroborated by data from Lindman et al [19] who found, using isothermal titration calorimetry technique, that particles with a diameter larger than 120 nm are covered with a dense monolayer of proteins.

To further characterize the particle-albumin interaction, we prepared dispersions with different particle and albumin concentrations. At a TiO_2 _(rutile) concentration of 0.02 mg/ml, HSA in a concentration higher than 0.015 mg/ml prevented the formation of coarse agglomerates. With the elevation of the HSA concentration in the dispersion, the average diameter of the particles increases in a concentration-dependent manner, obviously because the albumin layer on the particles was getting thicker. Further increase of the HSA concentration to 15 mg/ml results in saturation of the particle surfaces and the amount of free albumin molecules is getting high enough to be detectable. The lowest HSA/TiO_2 _(rutile) concentration ratio at which the particles are covered with HSA and the dispersion is stable was 0.75 (the calculated HSA/TiO_2 _(rutile) surface area ratio was 595). In this dispersion, there are ~33000 HSA molecules per each TiO_2 _(rutile) particle. This number is ~7 times higher than calculated to be necessary for a 100% HSA coverage of 200 nm spherical particles (4650 HSA molecules/particle) [19]. When we increase the TiO_2 _(rutile) concentration 10-fold and use the same HSA concentration, the number of HSA molecules is not high enough to completely cover TiO_2 _(rutile) particles and may not be sufficient to protect against agglomeration of particles. Consistent with this theory, we have seen coarse agglomerates in this dispersion. However, when we increased the TiO_2 _(rutile) and HSA concentrations equally (10-fold), letting the HSA/TiO_2 _(rutile) surface area ratio unchanged, the dispersion stayed stable. These data suggest that the required amount of stabilizer depends on the total surface area of the particles in the dispersion. According to the measurements with different TiO_2 _(rutile) concentrations, we choose 1.5 mg/ml HSA for our optimized method because this HSA concentration prevented the formation of coarse agglomerates in a concentration range that might be relevant for nanotoxicologic studies (0.002 mg/ml – 0.2 mg/ml).

If mouse serum was added at an amount (30 μl mouse serum to 1 ml dispersion) sufficient to achieve the albumin concentration similar to the dispersions prepared with HSA, it also prevented the formation of coarse agglomerates in TiO_2 _(rutile) dispersions (0.02 mg/ml). This amount of serum contains abundant proteins to saturate the particle surfaces as it has been reported that ~100 μl plasma saturates 1 mg 200 nm particles [20], that is 150 times less than that we used.

The TiO_2 _(rutile) particles prepared with HSA were stable for at least one week, whereas without stabilizer the particles started to form agglomerates in PBS. Similar results were obtained for other particles [7].

When nanoparticles get into the circulation they get in contact first with albumin and other serum proteins. These proteins cover the nanoparticles and form a protein corona [18]. Our optimized method uses also albumin or serum, thus nanoparticles dispersed with our method are covered with the same proteins nanoparticles encounter in the circulation.

We found that this optimized method was suitable for preparing dispersions without coarse agglomerates (average diameter < 290 nm) from nanosized TiO_2 _(rutile), ZnO, Ag, SWNT, MWNT, and diesel SRM2975 particulate matter. The polydispersity index value was high with SWNT, MWNT, and SiO_x_. The interpretation of the size parameter for nanotubes is different from that of other particles since nanotubes exhibit a different shape. In this case, size indicates the hydrodynamic diameter of a spherical particle that would move in the dispersion media with the same velocity as nanotubes. The high PdI of SWNT and MWNT nanotubes can also be attributed to the shape of these particles. In spite of the inaccuracy of the size data for measurements of nanotubes, dynamic light scattering gives important information about the agglomeration state of these dispersions. If nanotubes form agglomerates they move slower in the dispersion medium and the average diameter value is bigger than with well-dispersed nanotube dispersions. We found such a difference when we compared the dispersions prepared in PBS with dispersions prepared with our optimized method (see Table 1). For SiO_x _and TiO_2 _(anatase) particles, our method was less effective as indicated by a relatively high average diameter or PdI of particles. The zeta-potential value in PBS was always less negative than -30 mV suggesting that the electrostatic repulsive forces play a minor role for stabilization of these dispersions.

TEM confirmed our findings from dynamic light scattering as preparation with our optimized method resulted in an improved dispersion with any of the nanoparticles measured. The quantitative image analyses of the size of the TiO_2 _(rutile) particles yielded similar results to those from the dynamic light scattering measurements.

## Conclusion

**Figure 13:**
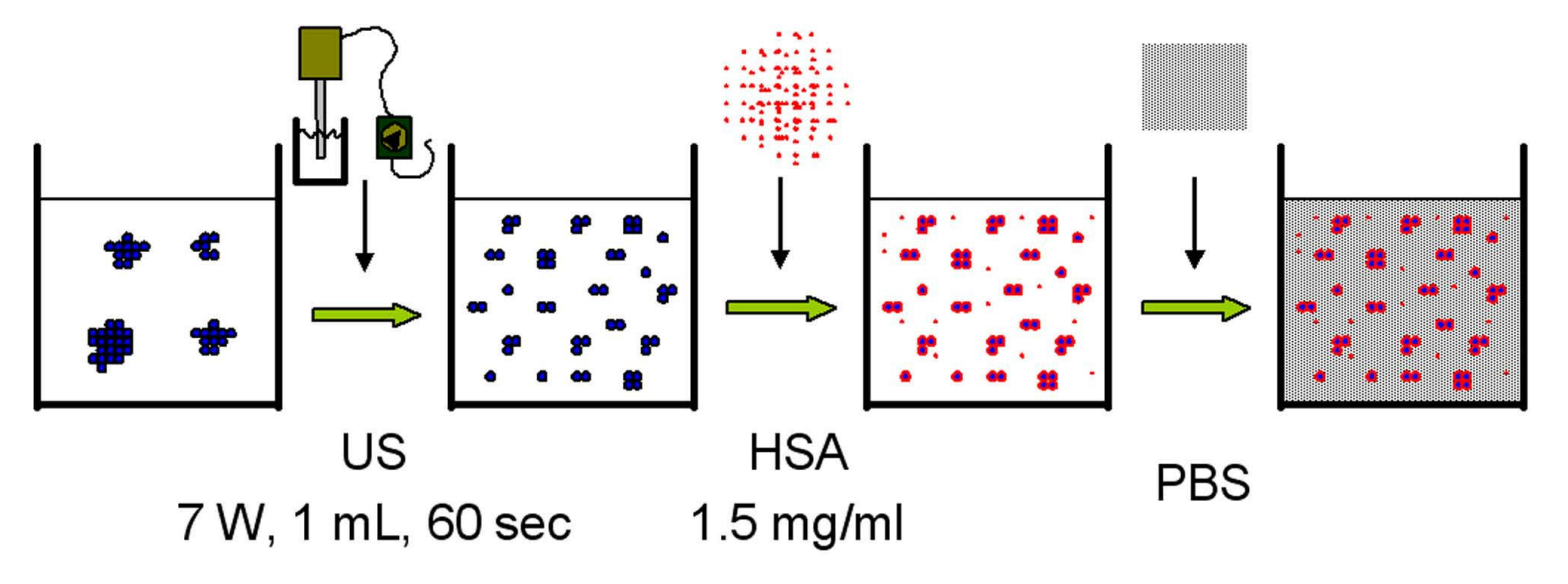
**Preparation steps of nanoparticle dispersion**. 1. Sonicate the nanoparticles in distilled water (power consumption: 7 W, 1 mL dispersion, 60 sec sonication, = 4.2 × 10^5 ^kJ/m^3^). 2. Add stabilizer (1.5 mg/ml HSA for dispersions with less than 0.2 mg/ml nanoparticle concentration or serum with a similar albumin concentration). 3. Add PBS to achieve physiological buffer and salt concentration.

We found that the following aspects are important to consider for the preparation of nanoparticle dispersions in physiological solutions (Fig. 13): *i*) the optimal sequence is first to sonicate the nanoparticles in distilled water, than to add the stabilizer, and finally to add buffered salt solution to the dispersion; *ii*) usage of a sonication energy high enough for deagglomerating the particles (>4.2 × 10^5 ^kJ/m^3^); *iii*) addition of albumin or serum as stabilizers at a concentration that is sufficient to cover the nanoparticles (1.5 mg/ml HSA for dispersions with less than 0.2 mg/ml nanoparticle concentration or serum with a similar albumin concentration).

In conclusion, the optimized dispersion method presented here appears to be effective and practicable for preparing dispersions of nanoparticles in physiological solutions without creating coarse agglomerates.

## Methods

### Materials

Titanium(IV) oxide nanopowder 99.5% rutile ~10 nm × 40 nm (TiO_2 _rutile), Titanium(IV) oxide nanopowder 99.7% anatase <25 nm (TiO_2 _anatase), Zinc oxide nanopowder <100 nm (ZnO), 10 × concentrated PBS, 10× concentrated RPMI 1640 medium bovine serum albumin (BSA) and mouse serum albumin (MSA) were purchased from Sigma-Aldrich, Schnelldorf, Germany. Human serum albumin (HSA) 50 g/L is from Baxter Deutschland Gmbh, Heidelberg, Germany. Plain (60 nm), carboxyl (60 nm) and amine modified polystyrene beads (65 nm) were purchased from Bangs Laboratories, Fishers, USA. S-purified single-wall nanotubes, outer diameter <2 nm length 1–5 μm (SWNT) and s-purified multi-wall nanotube, outer diameter 10–30 nm lengths 1–2 μm (MWNT) are from SES research, Houston, USA. SRM 2975 diesel particulate matter was purchased by the National Institute of Standards and Technology, Gaithersburg, USA. Silicon oxide 99.5%, 10 nm (SiO_x_) and silver synthesized 99.5+%, 30–50 nm (Ag) were ordered from the Nanostructured and Amorphous Materials Inc, Los Alamos, USA.

### Preparation of mouse serum

C57BL/6NCrl male mice (Charles River, Sulzfeld, Germany) were anesthetized with isoflurane-N_2_O (FiO_2 _0.35, 0.015 L/L isoflurane; Forene; Abbott GmbH, Wiesbaden, Germany). Blood was taken by heart puncture and allowed to clot. The blood was centrifuged with 5000 RPM for 20 minutes and the supernatant was taken. Serum samples were pooled and aliquots were stored in -20°C until use.

### Size distribution and zeta-potential measurement

The size distribution and the zeta potential of the nanoparticles were analyzed in aqueous dispersion with a Zetasizer-Nano ZS instrument (Malvern, Malvern Hills, United Kingdom).

Dynamic light scattering is used by the instrument to determine the size distribution of particles by measuring dynamic fluctuations of light scattering intensity caused by the Brownian motion of the particle. This technique yields a hydrodynamic diameter that is calculated *via *the Stokes-Einstein equation from the aforementioned measurements. The measurement gives as result the average hydrodynamic diameter of the particles, the peak values in the hydrodynamic diameter distribution and the polydispersity index (PdI) that describes the width of the particle size distribution. The PdI scale ranges from 0 to 1, with 0 being monodisperse and 1 being polydisperse. Each assigned size and PdI was the mean of 10 subruns. All measurements were carried out in triplicate with a temperature equilibration time of 1 minute at 25°C. The following parameters were used in the instrument settings to allow for a correct optical model: The R_i_(refractive index) values of the nanoparticles were taken from the literature. For the dispersant, a R_i _of 1.330 and a viscosity of 0.8872 cP were chosen. The data processing mode was set to high multi-modal resolution.

The measurement technique used by the Zetasizer Nano-ZS to measure the zeta potential of particles in a solution is known as phase analysis light scattering. This technique uses a laser, which is being passed through the sample, to measure the velocity of the particles in an applied electric field of a known value. The optical model for the zeta potential measurements was interpreted by the method of Smoluchowski since the particles were dispersed in polar solvents.

### Measurement of the effect of different ultrasound energies

A TiO_2 _(rutile) stock solution was prepared at a concentration of 0.02 mg/ml in distilled water. One ml of the nanoparticle dispersion was sonicated with a Hilscher UP50H 50 watt, 30 kHz sonicator (Hilscher Ultrasonics GmbH, Teltow, Germany) at different intensities (20%, 50% or 100%) and different durations of sonication (10 sec, 1 min or 5 min). The power consumption of the sonicator was measured with a power-measuring device during sonication of the particles (working operation) and during sonication of the air (no-load operation). The specific energy was calculated from the power consumption difference between working and no-load operation, from the duration of the sonication, and from the volume of the dispersion: E_spec_= (P_work_-P_no-load_) × time/volume [16].

### Preparation of the particle dispersions

For measuring the accuracy of size and zeta potential measurement, 60 nm plain, carboxyl and amine modified particles were prepared at a concentration of 0.02 mg/ml in distilled water.

To evaluate the different sequences of preparation steps, a TiO_2 _(rutile) stock solution was prepared at a concentration of 0.02 mg/ml in distilled water with or without sonication with 4.2 × 10^5 ^kJ/m^3 ^specific energy. Thirty μl of HSA (end concentration 1.5 mg/ml) or Tween 80 (end concentration 0.1%) was given to 870 μl of dispersion before or after the addition of 100 μl of a 10 × concentrated PBS solution.

The TiO_2 _(rutile) dispersion was also prepared in a similar way using RPMI 1640 cell culture medium and with the addition of other dispersion stabilizers, i.e. 1.5 mg/ml mouse serum albumin, 1.5 mg/ml bovine serum albumin, 0.1% Tween 80, or 30 μl mouse serum.

The effect of different TiO_2 _(rutile) and HSA concentrations was tested at TiO_2 _(rutile) concentrations of 0.002, 0.02, 0.2, 2 mg/ml and HSA concentrations of 0.0015, 0.015, 0.15, 1.5, and15 mg/ml.

The stability of 0.02 mg/ml TiO_2 _(rutile) dispersions made by sonication with 4.2 × 10^5 ^kJ/m^3 ^energy and addition of 1.5 mg/ml HSA followed by addition of PBS was measured for 1 week.

Dispersions were also made from TiO_2 _(anatase), ZnO, SWNT, MWNT, silver, SiO_x_, and SRM 2975 diesel nanoparticles by preparing a 0.02 mg/ml stock solution, sonicating with 4.2 × 10^5 ^kJ/m^3 ^specific ultrasound energy, adding of 1.5 mg/ml HSA, 0.1% Tween, or 30 μl serum prior to addition of concentrated PBS.

### Transmission electron microscopy

The particles of the dispersions were visualized with a transmission electron microscope (TEM Jeol JEM 2010) operated at 200 kV. Samples for TEM analysis were prepared by letting a drop of nanoparticle dispersion dry onto a holey carbon layer covered copper mesh grid (Agar Scientific Ltd., Stansted, UK). Mean particle size in the TiO_2 _(rutile) dispersion prepared with HSA was analyzed using Image J software (National Institutes of Health, Bethesda, USA) by measuring the diameter of 100 particles.

### Statistics

Data analysis was performed with a statistical software package (SigmaStat for Windows, Jandel Scientific, Erkrath, Germany). The ANOVA test followed by the Dunnett (comparison versus control), Student-Newman-Keuls (all pair wise comparison) were used for the estimation of stochastic probability. T-test was used to compare two groups. Mean values and standard deviation are given. *P *values < 0.05 were considered significant.

## Competing interests

The authors declare that they have no competing interests.

## Authors' contributions

PB designed the study, carried out the dynamic light scattering studies and drafted the manuscript. MV carried out the transmission electron microscopy. SS, MP participated in the dynamic light scattering measurements. AK, CR participated in the analyses of the data and were involved in drafting the manuscript. CC, TT, MR participated in the design of the study. FK conceived of the study, participated in its design and coordination, and helped to draft the manuscript. All authors read and approved the final manuscript.
